# Spatiotemporal distribution and population at risk of soil-transmitted helminth infections following an eight-year school-based deworming programme in Burundi, 2007–2014

**DOI:** 10.1186/s13071-017-2505-x

**Published:** 2017-11-23

**Authors:** Mohamad Assoum, Giuseppina Ortu, Maria-Gloria Basáñez, Colleen Lau, Archie C. A. Clements, Kate Halton, Alan Fenwick, Ricardo J. Soares Magalhães

**Affiliations:** 1 Children’s Health and Environment Program, Child Health Research Centre, The University of Queensland, Brisbane, Australia; 2 UQ Spatial Epidemiology Laboratory, School of Veterinary Science, The University of Queensland (Gatton Campus), Via Warrego Highway QLD, Gatton, 4343 Australia; 3 School of Medicine, The University of Queensland, Brisbane, Australia; 4 Schistosomiasis Control Initiative, Imperial College London, Department of Infectious Disease Epidemiology, School of Public Health, Faculty of Medicine (St. Mary’s Campus), Norfolk Place, W2 1PG, London, UK; 5 Present address: Malaria Consortium Headquarters. Development House, 56-64 Leonard Street EC2A 4LT, London, UK; 6 London Centre for Neglected Tropical Disease Research, Department of Infectious Disease Epidemiology, Faculty of Medicine (St. Mary’s Campus), Imperial College London, School of Public Health, Norfolk Place W2 1PG, London, UK; 7 Research School of Population Health, Australian National University, Canberra, Australia; 8 Queensland University of Technology, Brisbane, Australia

**Keywords:** Spatiotemporal modelling, Soil-transmitted helminths, Predictive risk mapping, Number of infections

## Abstract

**Background:**

Investigating the effect of successive annual deworming rounds on the spatiotemporal distribution of infection prevalence and numbers at risk for soil-transmitted helminths (STHs) can help identify communities nearing elimination and those needing further interventions. In this study, we aim to quantify the impact of an 8-year mass drug administration (MDA) programme (from 2007 to 2014) on the spatiotemporal distribution of prevalence of STH infections and to estimate the number of school-aged children infected with STHs in Burundi.

**Methods:**

During annual longitudinal school-based surveys in Burundi between 2007 and 2011, STH infection and anthropometric data for a total of 40,656 children were collected; these data were supplemented with data from a national survey conducted in 2014. Bayesian model based geostatistics (MBG) were used to generate predictive prevalence maps for each STH species and year. The numbers of children at-risk of infection per district between 2008 and 2014 were estimated as the product of the predictive prevalence maps and population density maps.

**Results:**

Overall, the degree of spatial clustering of STH infections decreased between 2008 and 2011; in 2014 the geographical clusters of all STH infections reappeared. The reduction in prevalence was small for *Ascaris lumbricoides* and *Trichuris trichiura* in the centre and central north of the country. Our predictive prevalence maps for hookworm indicate a reduction in prevalence along the periphery of the country. The predicted number of children infected with any STH species decreased substantially between 2007 and 2011, but in 2014 there was an increase in the predicted number of children infected with *A. lumbricoides* and *T. trichiura*. In 2014, the districts with the highest predicted number of children infected with *A. lumbricoides*, *T. trichiura* and hookworms were Kibuye district (*n* = 128,903), Mabayi district (*n* = 35,302) and Kiremba (*n* = 87,511), respectively.

**Conclusions:**

While the MDA programme in Burundi resulted in a reduction in STH prevalence, this reduction was spatiotemporally heterogeneous, with some pockets of high prevalence remaining, suggesting that treatment coverage and complementary interventions should be evaluated to improve impact.

**Electronic supplementary material:**

The online version of this article (10.1186/s13071-017-2505-x) contains supplementary material, which is available to authorized users.

## Background

Soil-transmitted helminth (STH) infections are intestinal nematode infections that affect approximately 1.6 billion people around the world, with the majority of infections occurring in resource-poor settings [1]. Since the signing of the London Declaration on Neglected Tropical Diseases (NTDs) in 2012, programmes for the control of STH infections and other NTDs have received renewed support from the pharmaceutical industry, the scientific community and key development agencies and stakeholders [2]. Reductions in prevalence of infection and associated morbidity can be achieved by successive mass drug administration (MDA). More recently, it has been argued that to further control and reach elimination targets, MDA campaigns would need to be integrated with water, sanitation and hygiene (WASH) programmes [3]. While MDA is seen as a cost-effective intervention to achieve morbidity control, rapid re-infection means that it can be ineffective at reducing transmission, especially for populations living perennially in STH-contaminated environments. Morbidity control through long-standing MDA programmes can be undermined by geographical disparities in drug coverage and drug efficacy and by socioeconomic conditions that limit the access and adequate utilisation of water and sanitation infrastructure [4].

The Schistosomiasis Control Initiative (SCI) has been actively involved with the planning, implementation and continued monitoring and evaluation of anthelmintic MDA programmes using albendazole (ALB) and mebendazole (MEB) in 16 sub-Saharan African (SSA) nations including Burundi. From 2007 until 2014, SCI supported an MDA programme in Burundi [5], primarily targeting school-aged children (SAC) and pregnant women. In 2007, a pilot longitudinal study was launched in 12 schools, followed in 2008 by an extension study, in which an additional 19 schools across the country were added [6]. The longitudinal study aimed to assess the impact of MDA on STH control in Burundi, and found that the overall prevalence of STH infection was statistically significantly reduced over the programme’s duration. However, this investigation also found that programmatic disruption (due to political and civil unrest in late 2009 through to 2010) resulted in substantially reduced levels of treatment coverage for that period, with a consequent detectable resurgence in STH prevalence. This highlighted the pressing need for STH control interventions to not rely solely on MDA, a strategy that may not be sustainable in the long term and which crucially requires achieving high levels of treatment coverage and adherence. This notion has been supported by numerous other studies [7–9], which indicate that in some endemic areas with high transmission, high intensity of infection may persist, requiring integration of MDA with WASH if elimination is to be achieved.

Predictive prevalence mapping based on spatial models that include environmental drivers of infection has been widely used to identify areas in SSA where communities are at highest risk of STH infection and thus deworming campaigns should be targeted [10–13]. Most studies have focused on estimating the spatial variation of indirect morbidity indicators, such as prevalence and intensity of infection [11, 14]. In the case of Burundi, predictive prevalence maps were produced in 2007 to focus treatment delivery based on areas of high uncertainty of high infection prevalence [15]. The study found that predictive prevalence mapping was indeed an effective tool for guiding MDA implementation to maximise deworming efficiency [5]. However, the impact of successive (annual) MDA rounds on the spatiotemporal variation of prevalence of STH infections such as the ensuing 8-year MDA programme in Burundi [6] has not been investigated. In our previous study, we found that disruption in the delivery of MDA, for example as a result of social unrest, may have contributed to the observed rebound in STH infection prevalence [6]. Furthermore, we found that the most common co-infections noted were *A. lumbricoides* and *T. trichiura* which peaked in 2008 at 2.72%. However rates of co-infections dropped substantially over the course of the MDA, with coinfections making up less than 2% per year following 2008. However, the impact of the MDA programme on the spatiotemporal distribution of prevalence of STH infection is largely unknown, and this understanding may have implications for the achievement of the overall intervention goal. Thus mapping heterogeneity in prevalence of infection over time is important, as it allows us to identify areas where MDA has been systematically successful and, more importantly, areas where it may have failed and where further MDA campaigns may be needed.

In the present study, we aim to: (i) quantify the impact of an 8-year MDA programme (from 2007 to 2014) on the geographical distribution of STH infection prevalence, and (ii) estimate the spatiotemporal variation in the number of STH-infected children following the 8-year programme. Our ultimate goal is to identify areas in Burundi where the impact of MDA has been systematically suboptimal at reducing prevalence and number of infections; this will help support the planning of further studies within these areas to understand the determinants of programme coverage and efficacy. Furthermore, it will also support the planning of further programmatic activities.

## Methods

### Data collection on STH infection

The protocol for data collection for the 2007–2011 surveys has been reported elsewhere [5]. In brief, the 2007–2011 surveys were conducted in conjunction with the delivery of the MDA programme. Data collected included child’s age, sex, height, weight, and parasite egg count by STH species. Stool samples were taken from 100 children (approximately 50 boys and 50 girls) per school [6]; each year, samples were collected in May and the MDA round was delivered in June. The diagnostic approach using the Kato-Katz method was detailed in our previous paper [6]. During the 2014 survey, similar data collection protocols comparable to those of the 2008–2011 period were used [6]. In 2014, all 12 schools from the pilot study plus 14 out of the 19 schools from the extension study were re-assessed to evaluate the prevalence and intensity of STH infection after 7 years of annual MDA [6]. In each school in 2014, 50 pupils aged between 12 and 16 years were recruited, with the exception of one pilot study school in which 100 pupils were recruited [5, 6]. In the 2008–2011 cohort, students were aged between 5 and 18 yrs. In 2014, the Ministry of Health, with the support of the Schistosomiasis Consortium for Operational Research and Evaluation (SCORE), conducted a national survey. Further details on the 2014 national survey have been reported elsewhere [6].

A single stool sample was collected from each child and duplicate slides were prepared [6]. Diagnosis of STH infection was performed using the Kato-Katz technique by trained local ground staff [16–18]. If a single egg of a given parasite species was found, the child was considered positive for that parasite species. Egg counts were used to detail the intensity of infection.

Geographical coordinates of each school were recorded using hand-held global positioning system (GPS) units. Overall prevalence of infection was calculated for each school and for each parasite species. These summary data were plotted in a geographical information system (GIS) (ArcMap version 10.3, ESRI, Redlands, CA, USA).

Infection data were gathered and collected from the same 31 schools during 3 years (2008, 2009 and 2011); however, due to civil unrest, only 12 out of the 31 were surveyed in 2010. In 2014, 26 out of the 31 schools were surveyed due to staffing issues. A total of 40,656 children were sampled over the 8 years. For the 2014 survey, height, weight and blood haemoglobin levels were not measured.

### Environmental and population data

Environmental influences on STH species, such as *A. lumbricoides* and *T. trichiura*, are well known. Land surface temperature (LST), soil type, and distance to a water bodies influence the survival of parasite eggs in the environment, and therefore determine the intensity of exposure [19]. Equally, the transmission of hookworm species is determined by climate and landscape, as their larvae burrow into the soil to survive in more favourable micro-environments [20]. Electronic data for a normalised difference vegetation index (NDVI) for a 30 × 30 m grid cell resolution were obtained from LandSAT 5 and 8 satellite images via the Google Earth Engine (GEE) database (Additional file 1: Table S1). Elevation data with a 30 × 30 m grid resolution, generated by a digital elevation model (DEM) from the Advanced Space-borne Thermal Emission and Reflection Radiometer (ASTER) Global Digital Elevation Model (GDEM), were obtained. LST data were also obtained from the ASTER system with a 500 × 500 m resolution. Precipitation data were collected from WorldClim with 1 × 1 km grid resolution. Remotely-sensed data for LST and NDVI were recorded monthly from 2007 to 2014 and a new annual raster file was created. The locations of large perennial inland water bodies were obtained from the Food and Agriculture Organization of the United Nations [21], and the distance to perennial inland water bodies (DPWB) was estimated for each survey location in the GIS. A 5 × 5 km resolution population density surface derived from the Global Rural-Urban Mapping Project (GRUMP) beta product was obtained from the Centre for International Earth Science Information Network (CIESIN) of the Earth Institute at Columbia University [22]. Values at each survey location for all environmental datasets were extracted in the GIS.

### Statistical analyses

#### Non-spatial models of STH infection

We assessed the temporal variation in environmental variables between 2007 and 2011, and it was found that the environmental variables did not vary significantly between years. As such, only the 2011 values were used for analyses (Additional file 1: Table S2). The relationship between the prevalence of infection with each parasite for each of the 31 schools and the arithmetic mean of each environmental variable at the school location was evaluated using scatter plots and lines of best fit. If the relationship was found to be linear, then the variable was included in the univariable and multivariable analysis as a fixed effect. Non-linear relationships were explored using linear regression; however, we did not consider any transformation for our final models. To identity the best set of uncorrelated predictor environmental covariates, the Pearson’s correlation coefficient was calculated for all pairs of environmental variables at all data locations for all years.

Fixed-effects binomial logistic regression models of prevalence of infection for each STH parasite species were developed in Stata version 10.1 (Stata Corporation, College Station, TX, USA). All univariable models included the individual-level variables age and sex as fixed effects and environmental covariates including either NDVI, LST, precipitation, DPWB or elevation. In the univariable analysis, Wald’s *P*-value of 0.2 was used to select variables to be included in the final multivariable models for each parasite species. Multivariable analysis was conducted including age and sex as fixed effects in the models and all selected environmental variables as fixed effects. Using a backward stepwise process of variable selection, variables with a *P*-value greater than 0.05 were excluded from the final multivariable model. However, if the coefficient of a given variable changed by more than one quarter of the value of the model preceding, due to the removal of the variable, then the removed variable was deemed to be a confounder and was retained in the final model. If a confounder was identified, the model with the lowest Akaike information criterion (AIC) was selected.

#### Analysis of residual spatial dependence

Residuals from the final multivariable models for each STH species were extracted for each survey year and residual spatial dependence was estimated using semivariograms, constructed using the *geoR* package of the statistical software R (The R Foundation for Statistical Computing) [23]. Semivariograms are defined by three parameters, namely the nugget, the range and the sill. The sill is constituted by the sum of the partial sill and the nugget. The partial sill and nugget correspond, respectively, to the components of residual variation that are spatially structured and unstructured variation (e.g. random error). The range indicates the average size of clusters of STH prevalence. The proportion of the variance in the data that is due to geographical location can be estimated by dividing the partial sill by the sill. A spatial trend in prevalence of infection is present when the sill of a semivariograms is not attained within a reasonable range, indicating the range is very large relative to the study area. Propensity for clustering is calculated by the partial sill divided by the sum of the partial sill and the nugget.

#### Spatial risk prediction and model validation

A total of 40,656 individual observations of STH infection status across all years were included in the analysis.

Spatial modelling was conducted on data collected between 2007 and 2011 and separately for 2014. Spatial prediction of STH prevalence was performed for each year using model-based geostatistics [24] with the Bayesian statistical software, OpenBUGS version 1.4 (Medical Research Council Biostatistics Unit, Cambridge, UK and Imperial College London, London, UK). All models included time, individual and environmental covariates as fixed effects plus a geostatistical random effect, in which spatial autocorrelation between locations was modelled using an exponentially decaying autocorrelation function. To improve identifiability and model convergence, all environmental variables were standardised by subtracting the mean and dividing by the standard deviation. The resulting regression coefficients for these variables represent the effect of a change of one standard deviation in these variables.

The outputs of Bayesian models, including parameter estimates and spatial prediction at unsampled locations, are distributions termed “posterior distributions”. The posterior distributions represent fully the uncertainties associated with the parameter estimates. We summarised the posterior distributions in terms of the posterior mean and standard deviation. Predicted prevalence estimates were categorised into 6 categories for visualisation: category 1 indicates very low STH prevalence (< 2%); category 2 indicates low prevalence (2–10%); category 3 indicates moderate STH prevalence (10–20%); category 4 indicates moderately-high prevalence (20–50%); category 5 indicates high prevalence (50–80%; and category 6 very high prevalence (> 80%). Prediction uncertainty was defined by the standard deviation and was categorised into 3 categories: low uncertainty (standard deviation < 0.2), moderate uncertainty (standard deviation 0.2–0.5) and high uncertainty (standard deviation > 0.5). Estimation of surface areas was conducted in ArcGIS using raster calculators and zonal statistics.

The predictive accuracy of the prevalence of infection models was assessed using the mean prediction error, the mean absolute error and the correlation coefficient between the predicted and observed values. The mean error quantifies the bias of the predictor, and the mean absolute error provides a measure for the association between the observed and predicted values. The correlation between the observed and predicted data was tested using Pearson’s correlation coefficient (Additional file 1: Table S3).

#### Estimation of number of school age children at risk of STH infection

Population density maps were multiplied by the predicted prevalence maps in ArcGIS version 10.3 (ESRI, Redlands, CA) to estimate the number of SAC predicted to be infected with each of the STH species per year per district. Population data for Burundi were obtained from CIESIN2000, and population growth rates for years 2005 to 2014 were obtained from the World Bank [25]. To estimate population for each survey year, the base population figure from 2011 was multiplied by the population growth rate.

## Results

### Dataset for analysis

All variables, with the exception of precipitation (for which a quadratic relationship was explored), had a linear relationship with STH infection prevalence. Precipitation was subsequently excluded from the final multivariable model because it was not statistically significantly associated with prevalence of infection. Initial univariate analyses demonstrated that LST and elevation were highly correlated, with a Pearson’s correlation coefficient of 0.9. However, the *P*-value and AIC scores for LST was lower than the *P*-value for elevation and for that reason elevation was excluded from the multivariable analysis. In the multivariate models, only LST and NDVI were found to be associated (*P* > 0.05) with the prevalence of all parasites at each survey location.

### Residual spatial variation

The residual semivariograms for *A. lumbricoides* prevalence of infection indicate that, after accounting for the effect of environmental covariates, infections were clustered during the years 2010 (average cluster size: 68 km; propensity for clustering: 80%) and 2011 (average cluster size: 77 km; propensity for clustering: 93%) (Additional file 1: Figure S1a-e). For *T. trichiura*, residual geographical clustering was present in 2008 (average cluster size: 52 km; propensity for clustering: 100%) and 2009 (average cluster size: 61 km; propensity for clustering: 100%) (Additional file 1: Figure S2a-e, Table S4). For hookworm infections, clustering was only found in 2008 and spatial trends in 2009 and 2010 (average cluster size: 22 km; propensity for clustering: 75%) (Additional file 1: Figure S3a-e, Table S4). In 2014, residual semivariograms for *A. lumbricoides* and hookworm prevalence demonstrated trends in spatial dependence, whilst no spatial dependence was evident for *T. trichiura*.

### Spatial risk prediction

**Fig. 1:**
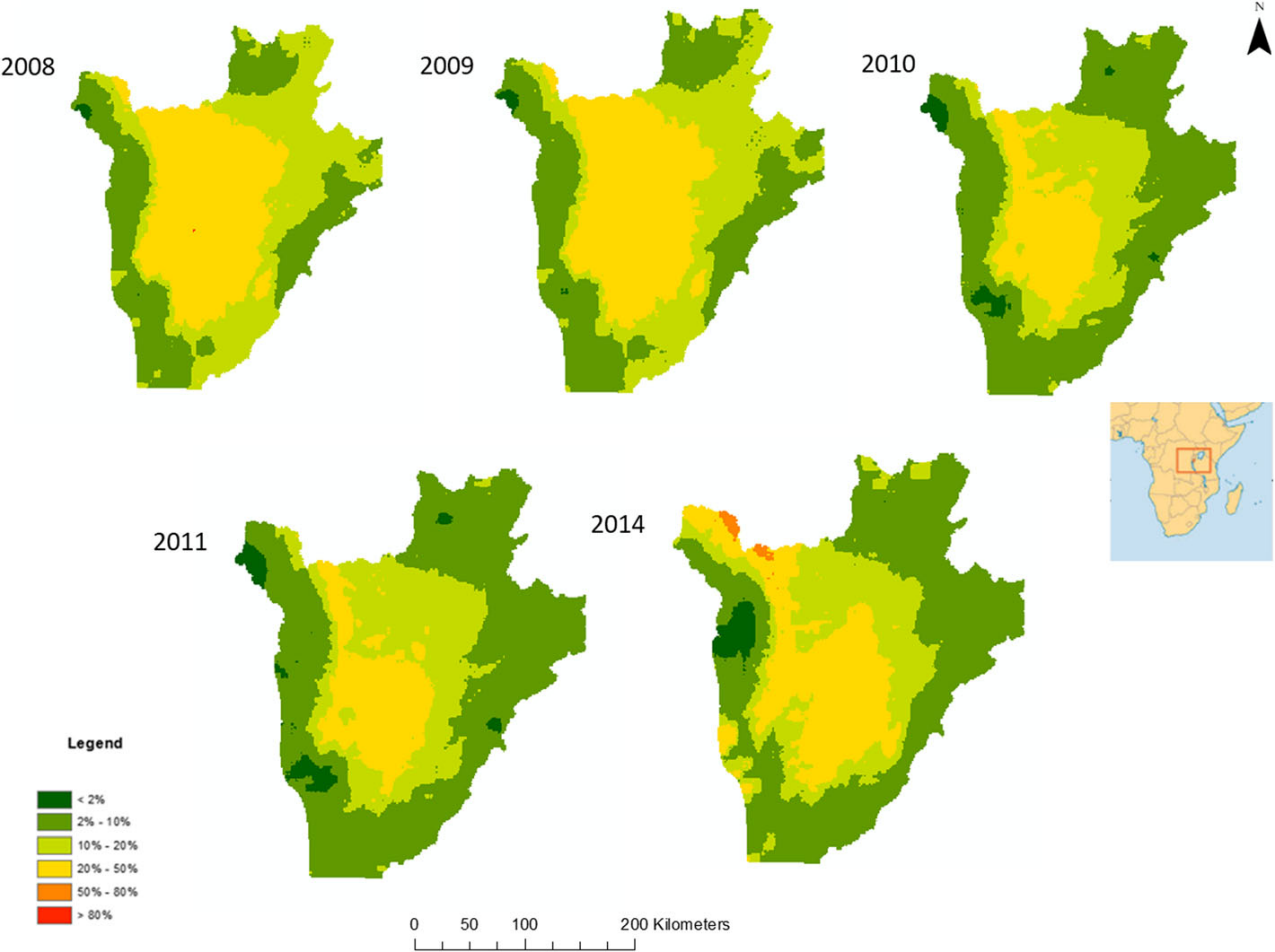
Predictive prevalence of infection maps for *A. lumbricoides*, 2008–2011 and 2014

**Fig. 2:**
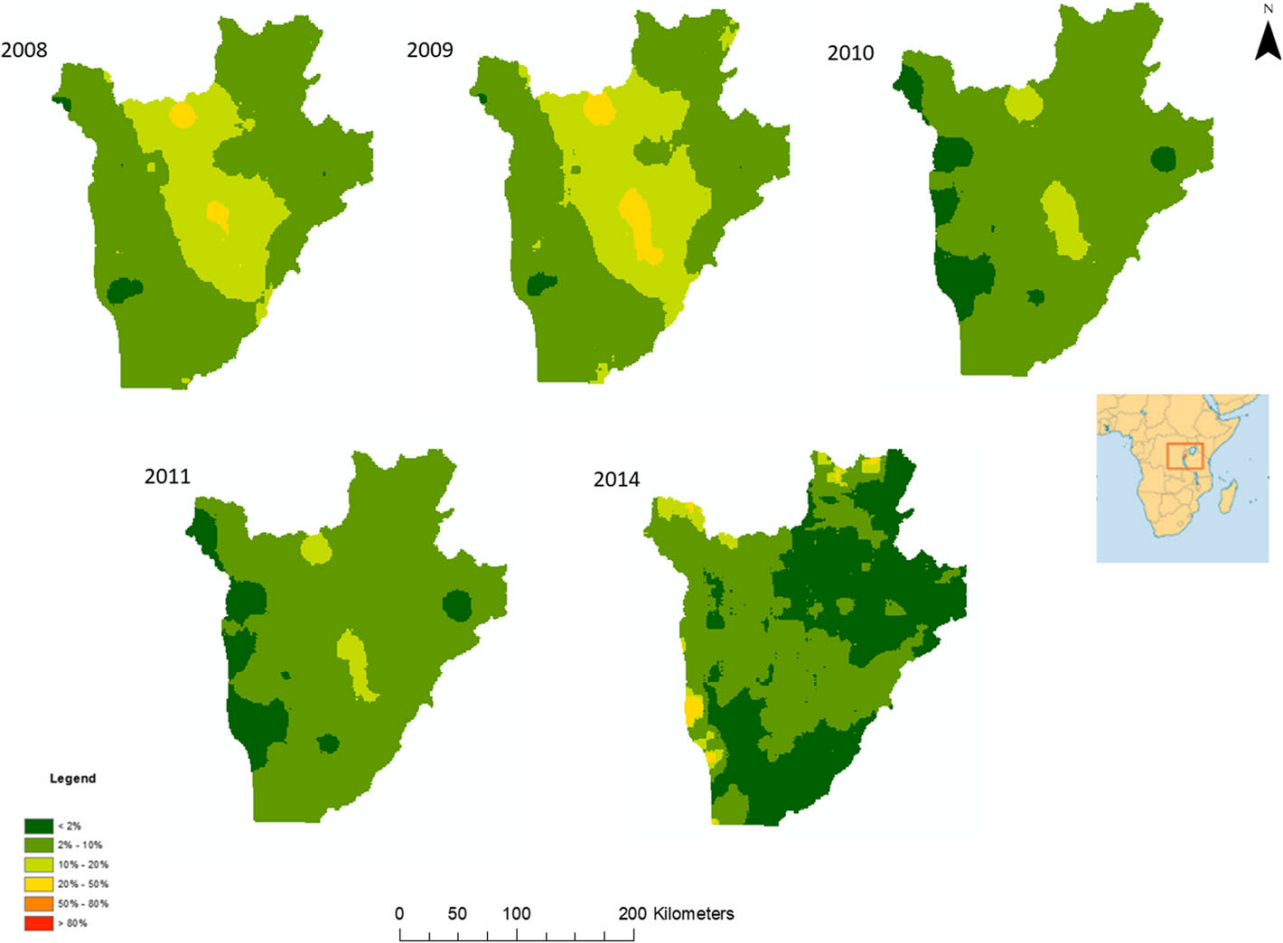
Predictive prevalence of infection maps for *T. trichiura*, 2008–2011 and 2014

**Fig. 3:**
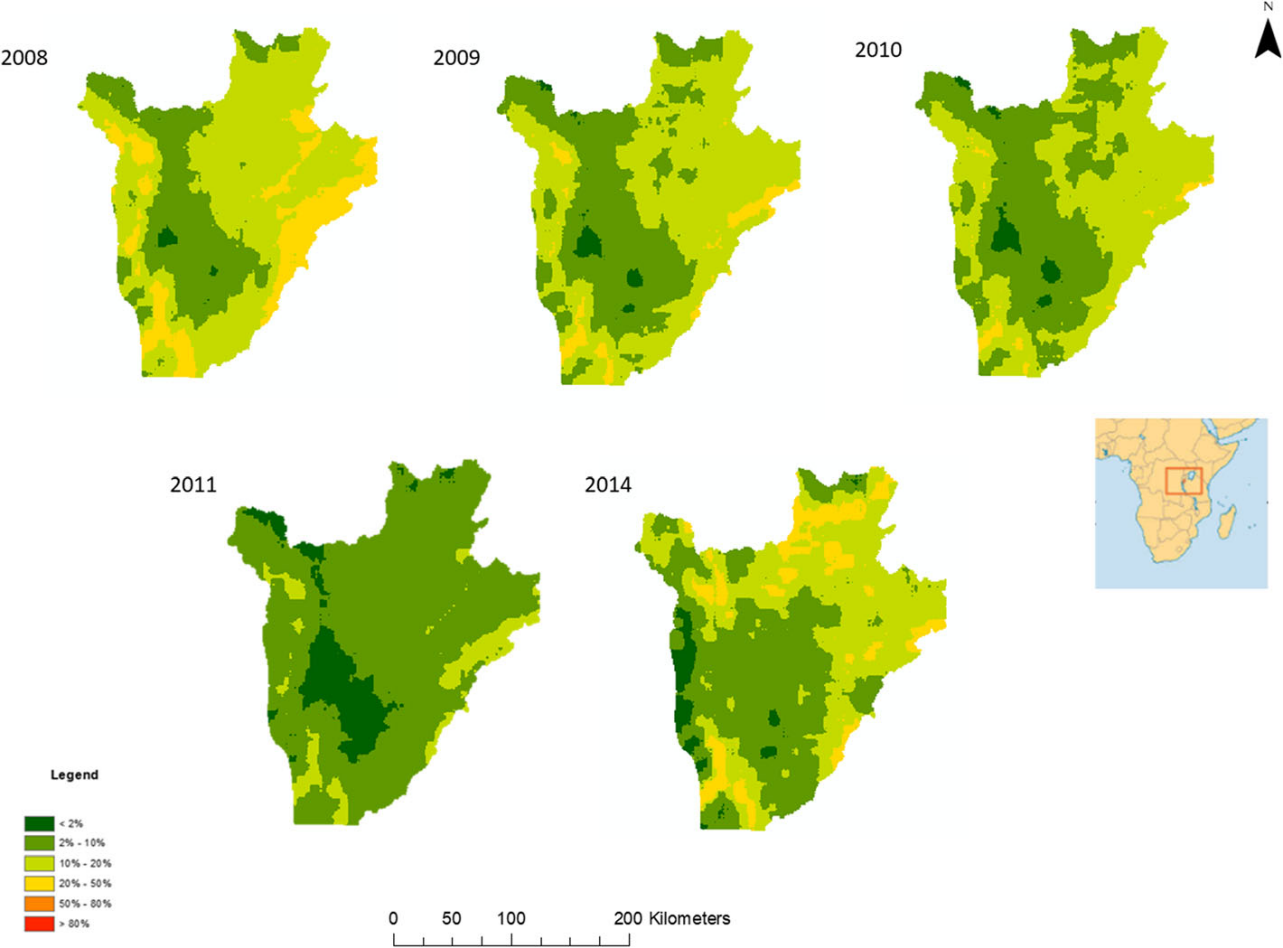
Predictive prevalence of infection maps for hookworm, 2008–2011 and 2014

Model effect sizes for each parasite between 2008 and 2011 and 2014 can be found in Additional file 1: Table S5. Predictive prevalence maps for both *A. lumbricoides* (Fig. 1) and *T. trichiura* (Fig. 2) demonstrate that the western region, the eastern border, the south-eastern border region and the north-eastern region of the country experienced a gradual reduction in STH prevalence from 2008 until 2014. Our predictive prevalence maps for *A. lumbricoides* show that between 2008 and 2014, the central south-western and north-western regions of the country areas demonstrated continued moderately high prevalence (> 20% and less than 50%) after several rounds of MDA were observed. Furthermore, areas to the north-west of the country experienced an increase in prevalence in 2014. Our predictive prevalence maps for *T. trichiura* show that in the central-northern region of the country there was a slight reduction in prevalence. This region, however, also maintained higher prevalence values (> 10% and less than 20%) than the surrounding regions; this is particularly evident between 2008 and 2011. In 2014, a small region where moderate prevalence (> 10% and less than 20%) of infection is predicted appeared in the south-western region of the country with a prevalence higher than in 2008. Our predictive prevalence maps for hookworm (Fig. 3) indicate that in 2008 the west and eastern regions had the highest predicted prevalence of infection (between 20 and 50%); by 2011 these regions observed a significant reduction in prevalence (predicted prevalence reaching 10–20%). However, in 2014 prevalence of hookworm infection was predicted to be as high as 50% in the north southwest and small pockets in the east of the country.

For all parasite species, there was a substantial reduction in the total surface area of high and moderate prevalence categories between 2008 and 2011 with a resurgence in 2014 (Table 1). For all parasites our results indicate a decrease in the overall surface area of moderate and high prevalence categories from 15,734 m^2^ in 2008 to 4277 m^2^ in 2011. The results for *A. lumbricoides* demonstrate a reduction in the surface areas for high infection prevalence (> 50% and less than 80%) and moderate infection prevalence (> 20% and less than 50%) between 2008 and 2011 and an increase in 2014, with an overall total surface area of 10,310 km^2^ in 2008, 4277 km^2^ in 2011, and 6802 km^2^ in 2014. These changes were accompanied by a substantial increase in surface area of regions within the low infection prevalence category. For *T. trichiura*, there was a reduction in surface area for high (> 50% and less than 80%) and moderately high infection prevalence (> 20% and less than 50%) from 416 km^2^ in 2008 to 0 km^2^ in 2011 and an increase of 286 km^2^ in 2014. For *T. trichiura* there were no very high prevalence categories (> 80%) from 2008 to 2014, with all high prevalence areas (> 50% and less than 80%) transitioned to a moderately high prevalence (> 20% and less than 50%) status. Nearly all moderate prevalence categories (> 10% and less than 20%) transitioned into low prevalence (> 2% and less than 10%) categories. In 2008, very few areas were classified with very low prevalence (< 2%) (surface area 346 km^2^); however, by 2014 most areas in the country were classified with very low prevalence (surface area 13,006 km^2^). For hookworm there was a substantial decline in moderate prevalence surface area between 2008 and 2011, from 4646 km^2^ to 0 km^2^; however, a resurgence of the moderate and high prevalence categories was evident in 2014, with a total surface area of 3079 km^2^.

**Table 1:** Changes in surface area (in km^2^) of prevalence of infection categories in Burundi for 2008–2011 and 2014

	2008	2009	2010	2011	2014
Prevalence of *A. lumbricoides* infections					
< 2%	78	130	586	791	605
2–10%	8348	9510	15,017	15,404	13,214
10–20%	9104	8799	7113	7370	7046
20–50%	10,311	9404	5127	4277	6802
50–80%	2	0	0	0	176
> 80%	0	0	0	0	0
Prevalence of *T. trichiura* infections					
< 2%	346	231	2634	3189	13,006
2–10%	19,844	17,679	23,901	23,804	13,730
10–20%	7237	8915	1307	849	821
20–50%	416	1018	0	0	286
50–80%	0	0	0	0	0
> 80%	0	0	0	0	0
Prevalence of hookworm infections					
< 2%	165	491	701	3223	1061
2–10%	7770	10,890	12,908	22,344	12,305
10–20%	15,262	15,299	13,757	2276	11,397
20–50%	4646	1162	476	0	3079
50–80%	0	0	0	0	0
> 80%	0	0	0	0	0

For *A. lumbricoides,* regions in the north, south and east of the country showed low to very low prediction uncertainty. Low (standard deviation below 0.2) to moderate (standard deviation between 0.2–0.5) uncertainty was evident in the central and western regions of the country (Additional file 1: Figure S4). Predictions for *T. trichiura* had low to very low uncertainty throughout the country. Patches of low to moderate uncertainty were evident in the centre of the country between 2008 and 2011, whilst in 2014 moderate uncertainty corresponded closely to areas of moderate prevalence of infection (Additional file 1: Figure S5). For hookworm, uncertainty was low across the country between 2008 and 2011. However, in 2014, moderate uncertainty was evident in the northern, eastern and southern regions of the country (Additional file 1: Figure S6).

### Model validation

The models for *A. lumbricoides* prevalence demonstrated low mean absolute error (MAE) for all years (ranging between 0.03 and 0.06) with high Pearson’s correlation coefficients (PCC) (ranging between 0.84 and 0.98) for all years (Additional file 1: Table S3). The models for *T. trichiura* prevalence demonstrated low mean absolute error for all years (MAE between 0.01 and 0.04) with high Pearson’s correlation coefficients (ranging between 0.93 and 0.94) for 2008, 2009 and 2011. Correlation was weak in 2010 and 2014, ranging between 0.16 and 0.47 (Additional file 1: Table S3). The models for hookworm prevalence also demonstrated low mean absolute error (ranging between 0.03 and 0.04) and high Pearson’s correlation coefficients (ranging between 0.74 and 0.83) for all years (Additional file 1: Table S3).

### Spatial heterogeneity in the number of school age children infected with STH

**Fig. 4:**
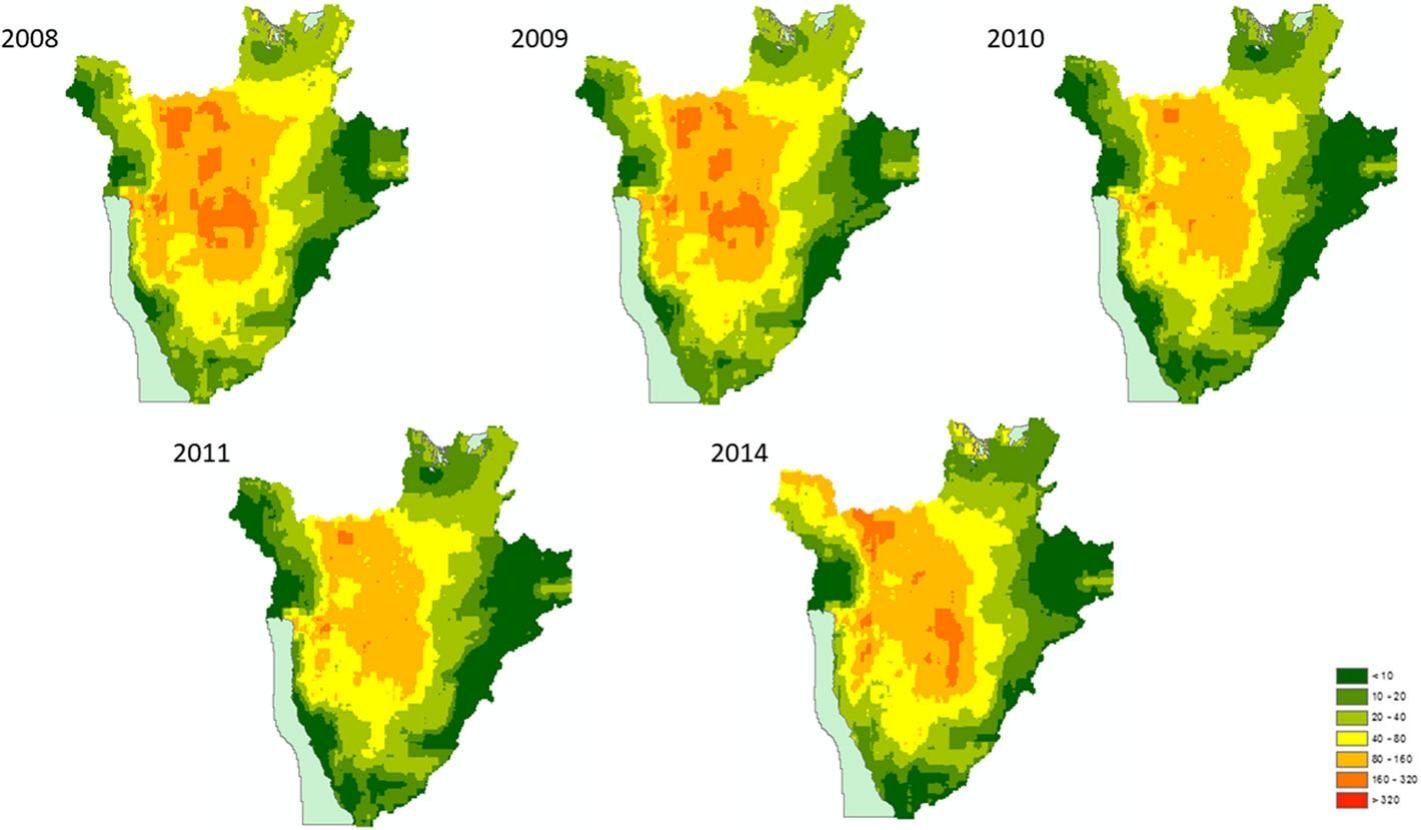
Predicted number of children aged 5 to 15 infected with *Ascaris lumbricoides* in 2008–2011 and 2014

**Fig. 5:**
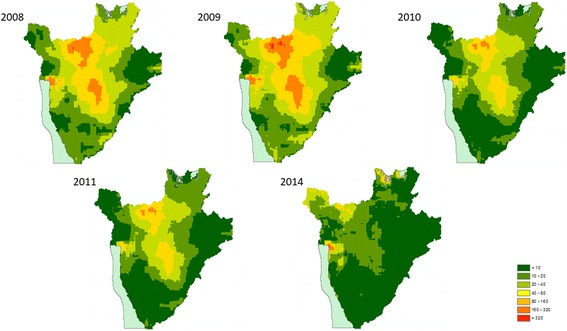
Predicted number of children aged 5 to 15 infected with *Trichuris trichiura* in 2008–2011 and 2014

**Fig. 6:**
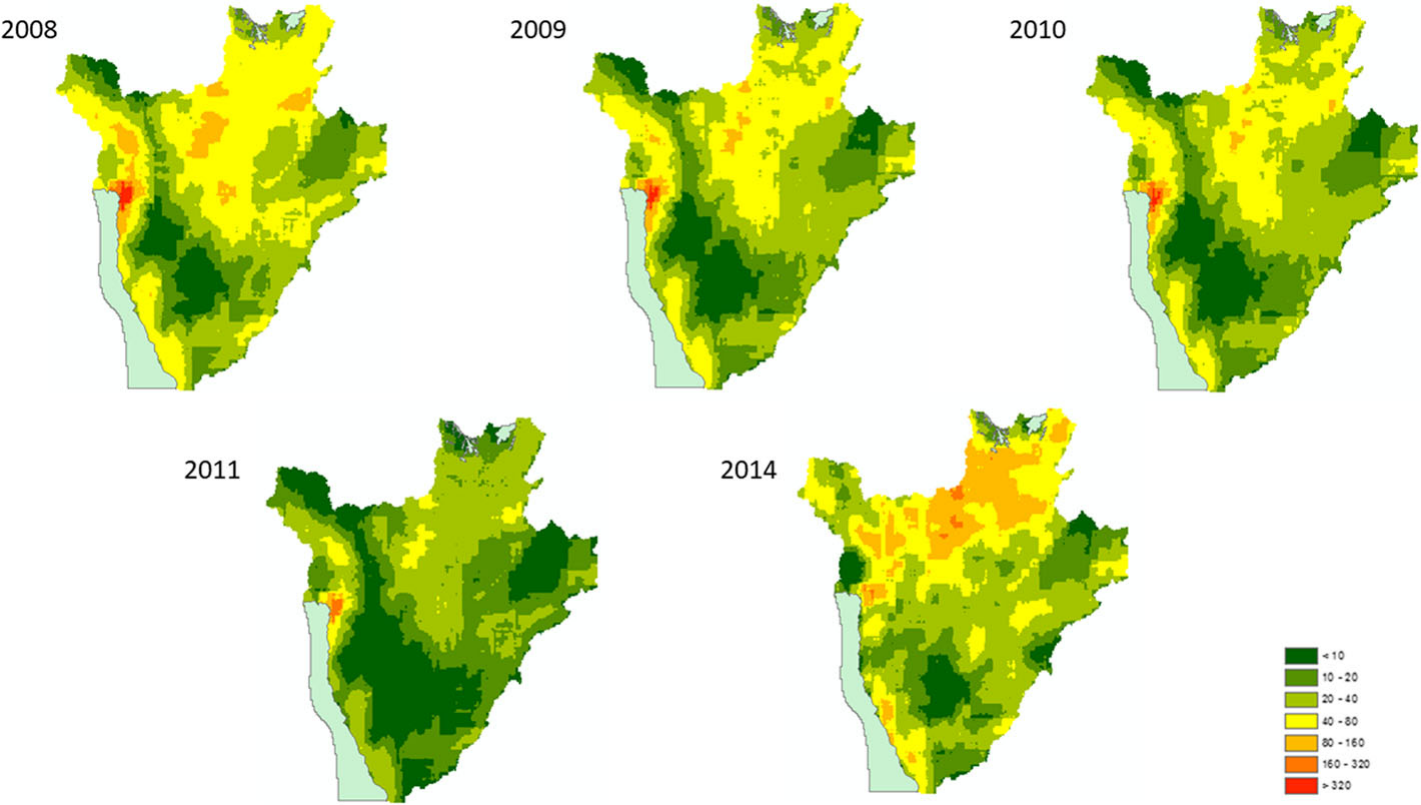
Predicted number of children aged 5 to 15 infected with hookworm in 2008–2011 and 2014

An overall reduction in the number of infected SAC was evident for all parasite species from 2008 to 2011 in all districts. In 2014, an estimated total of 4,098,816 children were infected with at least one species, either *A. lumbricoides* (Fig. 4), *T. trichiura* (Fig. 5) or hookworms (Fig. 6). A reduction in the predicted number of children infected with *A. lumbricoides* was evident from 2008 to 2011, with the highest predicted number of infected children in 2008 being 119,619 infected children in the Gitega district for *A. lumbricoides* and in 2014 in the Kibuye district, with 128,903 children infected. For *T. trichiura*, and in 2008, the district with the highest number of infected children was Ngozi, with 65,669 infected children. In 2014, the Mabayi district was predicted to have the highest number of SAC with *T. trichiura*, with 35,302 infected children. In 2008, hookworm infection was highest in the Muyinga district, with an estimated 66,828 children infected with *N. americanus/A. duodenale*. In 2014, this figure increased to 87,511 in Kiremba. Overall, the number of children infected with hookworm saw a 4.9% increase between 2008 and 2014 (Additional file 1: Table S6).

## Discussion

In our previous study we reported marked variation in STH prevalence between the different years of the Burundian MDA programme, thus justifying a more formal assessment of the spatiotemporal distribution of STH prevalence in Burundi [6]. Here we quantify, for the first time, the impact of an 8-year MDA programme on the spatiotemporal variation in infection prevalence and predict the number of children infected with each STH species over the course of the programme. The maps and infection burden estimates presented here can help intervention planning to best utilise resources to ensure that areas that are most at risk of STH infection are targeted [10, 26–28]. Our maps could also be useful to guide the control programme in Burundi on how to best reach transmission control and elimination goals by linking with transmission dynamics models [29].

The effect of socio-economic, climate and physical environment on STH infections is well known [19, 30] and has been used to investigate the spatial distribution of STH infections [20, 31, 32]. In our multivariable models of prevalence of *A. lumbricoides* and *T. trichiura* infections, we found that areas with higher prevalence of infection were associated with temperatures between 25 and 37 °C and lower vegetation indexes. This is in line with existing evidence indicating that *A. lumbricoides* and *T. trichiura* species require temperatures below 37 °C and low NDVI values to facilitate their survival and transmission [19].

### Spatiotemporal variation in STH infection prevalence 2008–2014

Quantifying the relative change in the geographical clustering in different parasite species over the duration of successive annual MDAs can help determine the population effect of anthelmintic treatment and the likelihood of particular areas achieving elimination. It is expected that, as a result of MDA, clusters of high prevalence of infection will be reduced in size as areas less resistant to treatment shift their level of endemicity to moderate and low prevalence of infection. As a result of successive MDA rounds between 2008 and 2010, prevalence of STH infection was reduced [6]; the results of the present study demonstrate that the observed reduction in prevalence between 2009 and 2011 was accompanied by a concurrent reduction in the geographical clustering of STH infections, particularly for *T. trichuris* and hookworm infections, as evidenced by a reduction in the propensity for clustering (from 2009 to 2014 in the case of both species). This finding is corroborated by our predictive prevalence maps which indicate a reduction in the prevalence of *T. trichiura* infection in central districts of the country and along the periphery of the country in the case of hookworm where these infections where principally distributed.

This suggests that during 2008–2011 geographical patterns of *T. trichiura* and hookworm infections shifted from widespread high-endemicity clusters into less defined prevalence clusters but still exhibited some residual spatial trend in infection. In the case of *A. lumbricoides*, despite the reduction in prevalence of infection, the spatial patterns from 2008 to 2011 remained relatively stable, with highly endemic areas present in the central districts.

However, resurgence in prevalence of infection was detected in 2014 in the northwest and southwest regions of the country for *A. lumbricoides* and *T. trichiura* and in the northwest, southwest, east and northeast for hookworm. This increase resulted in the re-emergence of the moderate infection prevalence category, with *A. lumbricoides* also experiencing resurgence in the high prevalence category. The precise reasons for this resurgence are largely unknown since the longitudinal study concluded in 2011 and follow-up surveys were not conducted until 2014.

Areas of civil unrest were mainly documented in the western, south-western, north-western and north-eastern regions of the country [33, 34]. The original 12 pilot school sites (situated mainly in the west of the country [6]) remained relatively safe, while in all extension survey sites (more evenly distributed throughout the country [6]), treatment was halted in 2010 due to disruption by the civil unrest. Disruption of the MDA programme in affected areas is likely to have impacted on the spatial distribution of infections due to uneven coverage of MDA. Central and eastern areas received the majority of the internally-displaced population and there was a notable division between treated and untreated populations there. The 12-month treatment disruption and the observed impacts highlight the potential impact of population dynamics and contextualising population movement in the context of STH transmission; the importance of developing spatially-structured dynamic models in addition to spatially-structured geostatistical models; and the need to develop WASH infrastructure that would change prevailing transmission conditions more sustainably.

The above regions’ higher initial prevalence rates, for example in Kibumbu, Gitega and their immediate districts [6], could also be a factor in resurgence. Even currently hypo-endemic areas may have an increased risk of resurgence or reintroduction if they were formerly hyper-endemic, thus emphasizing that MDA programmes alone are not sustainable in maintaining low morbidity in the long term in areas prone to destabilisation. Moreover, the overall geographical distribution of hookworm species appeared to be inversely associated with that of *A. lumbricoides* and *T. trichiura*. This, together with the fact that the different STH species are characterised by different age profiles of infection, highlights the need to understand the macro- and micro-epidemiology of the STH component infections separately.

### Spatial variation in treatment needs following 8-year MDA in Burundi

Combining infection prevalence maps with estimates of population numbers has allowed us to: (i) estimate the temporal variation in the predicted number of infected SAC over different years of the MDA programme in Burundi; (ii) identify areas where reductions in these numbers were more or less pronounced and, therefore, to highlight areas where the number of infected SAC remained roughly unaltered; and (iii) predict geographically the number of SAC infected for 2014, the year during which a “national reassessment” of the programme was conducted. By taking population density into account, our results demonstrate that in the case of *A. lumbricoides* and *T. trichiura*, the central and central northern regions of Burundi should be the focus of future MDA programmes, as these contain communities where the number of infected children is predicted to be highest. However, in the case of hookworm infection, the eastern western region as well as the northern regions should be of particular focus. Predictive infection distribution maps are an important extension that allow for effective and programmatically helpful decision-support tools to target treatments to populations in greatest need. An important extension to our work could involve coupling our models to dynamic disease transmission models that account for internal population migration/displacement.

### Limitations

A number of limitations need to be considered when interpreting our results. First, our results indicated that areas of moderate uncertainty are co-distributed with areas of moderate to high prevalence. This may be so because our data had few cases of moderate and heavy infections from 2009 onwards. One of the principal purposes of evaluating the level of uncertainty in mapped outputs is to demonstrate areas where further investigations are needed [35]. Second, the presence and intensity of STH infections are determined by poor hygiene and sanitation, and socio-economic demographics [3, 36, 37], but data indicating the state of hygiene practices and the availability of sanitation infrastructure in the study districts were not available. Third, although we endeavoured to obtain remotely-sensed data with the highest possible resolution, in some instances, the resolution of the data was not ideal (with pixels approximating 1 km by 1 km). This is a limiting factor as it contributes to regression dilution bias. Similarly, population maps used in our models have been adjusted using general annual growth rates and as such they are subject to accuracy issues as annual growth rates may have not been necessarily homogenous across the entire nation. Fourth, we did not account in our modelling framework for the impact of other NTD interventions, such as treatment of onchocerciasis, which not only is community wide (rather than targeted at particular age and population groups), but also includes ivermectin, an anthelmintic which, when combined with ABZ, has a better efficacy for *T. trichiura* than ABZ or MBZ on their own [29]. This gap may act as a critical factor influencing the differences observed between 2011 and 2014, but it was difficult to obtain programmatic data for these two programmes and the extent of their overlap with the STH programme. Finally, while our validation statistics demonstrate high correlation and low mean errors for most parasite species and years, this was not the case for *T. trichiura* in 2010 and 2014, where Pearson’s correlation coefficient was poor (i.e. < 0.7). This is likely due to the fact that more than 30% of the survey locations had no *T. trichiura* infections for the target age and sex subpopulation of our prediction model.

## Conclusions

Follow-up parasitological surveys, as well as MBG mapping updates throughout the programme, have been used to monitor the overall progress achieved with the STH MDA intervention in Burundi from 2007 to 2014 in terms of changes in the spatiotemporal clustering of prevalence, surface area of endemicity levels and numbers of children at risk. Together with a decrease in prevalence, a decrease in infection clustering was also observed, suggesting that successive MDA rounds were successful at reducing infection clusters [38], shifting infection patterns from clusters of high to moderate infection levels to more dispersed cases of infection. This was evident for all parasite species over the course of the MDA programme. Furthermore, the small-scale geographical distribution of STH species also changed over the course of this programme. The number of infected SAC varied geographically across the years and for the different parasite species. Finally, the success of the MDA programme appears to be very sensitive to perturbations to the programme and possibly to internal migration with and areas rebounding to higher prevalence levels in a matter of a couple of years.

## Additional file

Additional file 1:
**Table S1.** Environmental data summary. **Table S2.** Correlation coefficients for NDVI and LST, 2007–2011. **Table S3.** Model validation results, mean prediction error, absolute prediction error and Pearson’s correlation coefficient for all parasites, 2008–2011 and 2014. **Table S4.** Average size of clusters and propensity of clustering per parasite per year. **Table S5.** Model effect sizes for all parasites, 2008–2011 and 2014. **Table S6.** Total number of infected children per year per parasite 2008–2011 and 2014. **Figure S1.** Residual semivariograms for prevalence of infection with *Ascaris lumbricoides* in Burundi for years 2007–2011 and 2014. **Figure S2.** Residual semivariograms for prevalence of infection with *Trichuris trichiura* in Burundi for years 2007–2011 and 2014. **Figure S3.** Residual semivariograms for prevalence of infection with hookworm in Burundi for years 2007–2011 and 2014. **Figure S4.** Posterior mean standard deviation of predicted prevalence of infection with *Ascaris lumbricoides* in Burundi for 2008–2011 and 2014. **Figure S5.** Posterior mean standard deviation of predicted prevalence of infection with *Trichuris trichiura* in Burundi for 2008–2011 and 2014. **Figure S6.** Posterior mean standard deviation of predicted prevalence of infection with hookworm in Burundi for 2008–2011 and 2014. (DOCX 761 kb)

## Abbreviations

95% CI: 95% confidence interval
ABZ: Albendazole
AIC: Akaike information criterion
ASTER: Advanced spaceborne thermal emission and reflection radiometer
AUC: Area under curve
CIESIN: Center for International Earth Science Information Network
DEM: Digital elevation model
DPWB: Distance to perennial water body
GDEM: Global digital elevation map
GEE: Google Earth Engine
GIS: Geographical information systems
GLM: Generalised linear models
GPS: Global positioning system
GRUMP: Global rural urban mapping project
LST: Land surface temperature
MAE: Mean absolute error
MBG: Model-based geostatistics
MBZ: Mebendazole
MDA: Mass drug administration
NDVI: Normalized differential vegetation index
NTD: Neglected tropical diseases
PCC: Pearson’s correlation coefficient
ROC: Receiver operating characteristic
SAC: School-aged children
SCI: The Schistosomiasis Control Initiative
SCORE: Schistosomiasis Consortium for Operational Research and Evaluation
SSA: Sub-Saharan Africa
STH: Soil-transmitted helminths
WASH: Water and sanitation and hygiene
WHO: World Health Organization
