# Supplementary material for: Spatiotemporal distribution and population at risk of soil-transmitted helminth infections following an eight-year school-based deworming programme in Burundi, 2007–2014
Source: Parasit Vectors. 2017 Nov 23;10:583. doi: 10.1186/s13071-017-2505-x (PMC5701347; doi:10.1186/s13071-017-2505-x)
Supplement: Additional file 1: — Table S1. Environmental data summary. Table S2. Correlation coefficients for NDVI and LST, 2007–2011. Table S3. Model validation results, mean prediction error, absolute prediction error and Pearson’s correlation coefficient for all parasites, 2008–2011 and 2014. Table S4. Average size of clusters and propensity of clustering per parasite per year. Table S5. Model effect sizes for all parasites, 2008–2011 and 2014. Table S6. Total number of infected children per year per parasite 2008–2011 and 2014. Figure S1. Residual semivariograms for prevalence of infection with Ascaris lumbricoides in Burundi for years 2007–2011 and 2014. Figure S2. Residual semivariograms for prevalence of infection with Trichuris trichiura in Burundi for years 2007–2011 and 2014. Figure S3. Residual semivariograms for prevalence of infection with hookworm in Burundi for years 2007–2011 and 2014. Figure S4. Posterior mean standard deviation of predicted prevalence of infection with Ascaris lumbricoides in Burundi for 2008–2011 and 2014. Figure S5. Posterior mean standard deviation of predicted prevalence of infection with Trichuris trichiura in Burundi for 2008–2011 and 2014. Figure S6. Posterior mean standard deviation of predicted prevalence of infection with hookworm in Burundi for 2008–2011 and 2014. (DOCX 761 kb) [file 13071_2017_2505_MOESM1_ESM.docx]

**Additional file 1**

Figure S1: Residual semivariograms for prevalence of infection with *Ascaris Lumbricoides* in Burundi for years 2007 – 2011 and 2014.


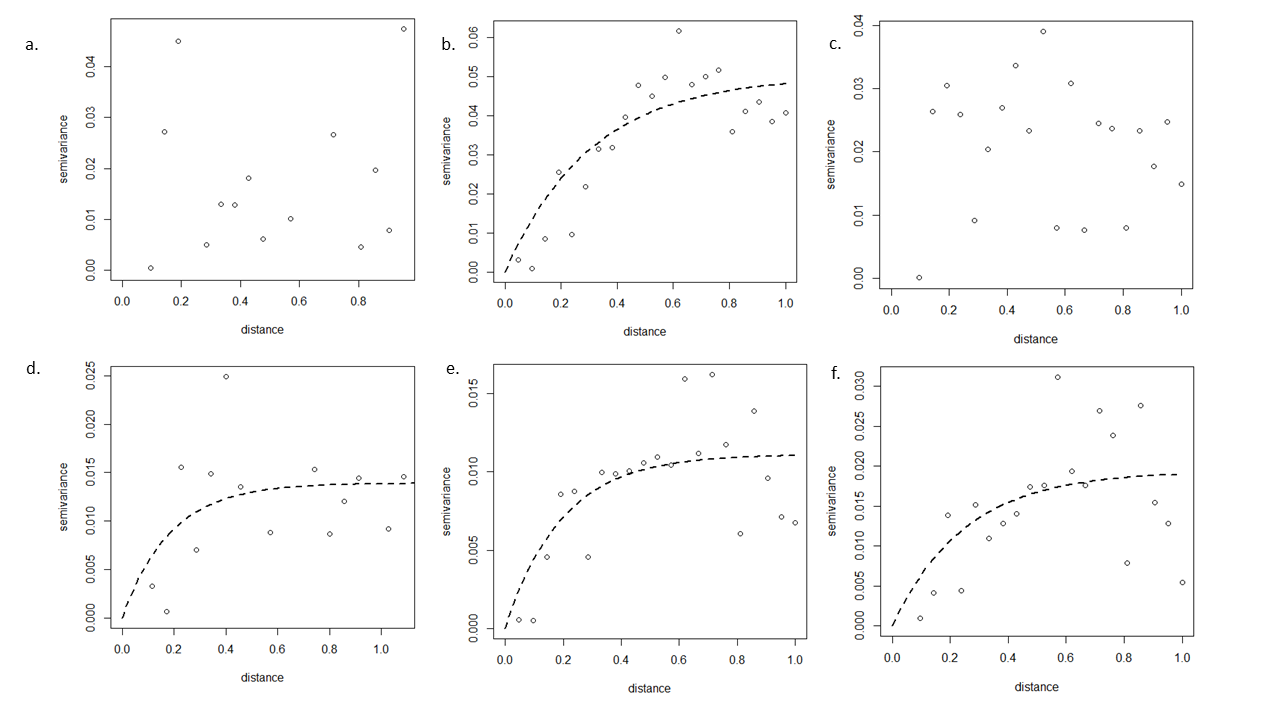


Figure S2: Residual semivariograms for prevalence of infection with *Trichuris trichiura* in Burundi for years 2007 – 2011 and 2014.


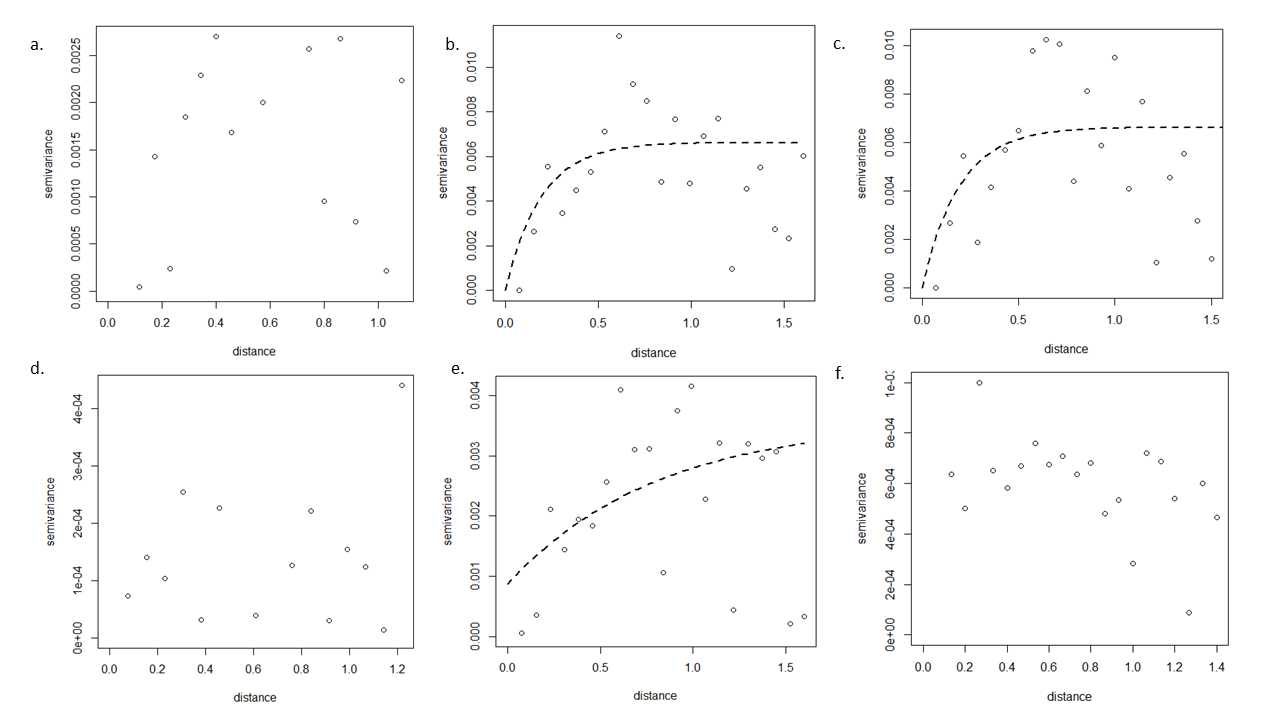


Figure S3: Residual semivariograms for prevalence of infection with *hookworm* in Burundi for years 2007 – 2011 and 2014.


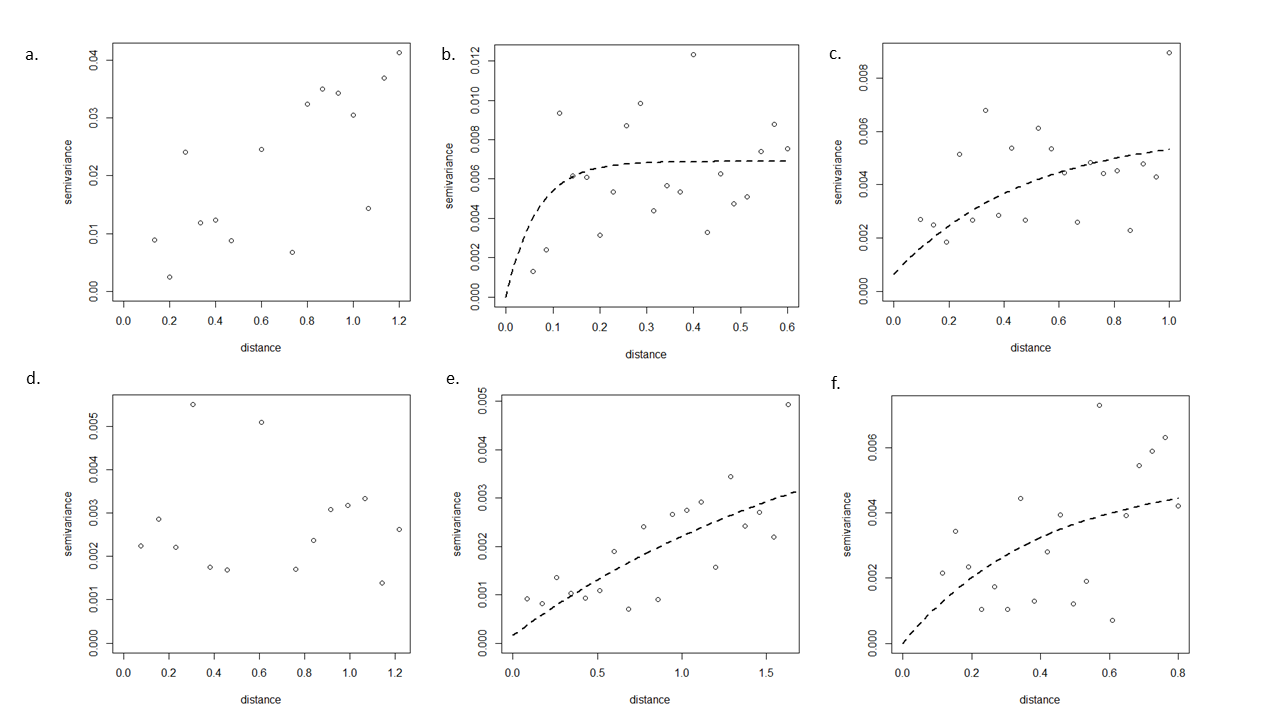


Figure S4: Posterior mean standard deviation of predicted prevalence of infection with Ascaris lumbricoides in Burundi for 2008 – 2011 and 2014


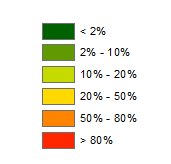

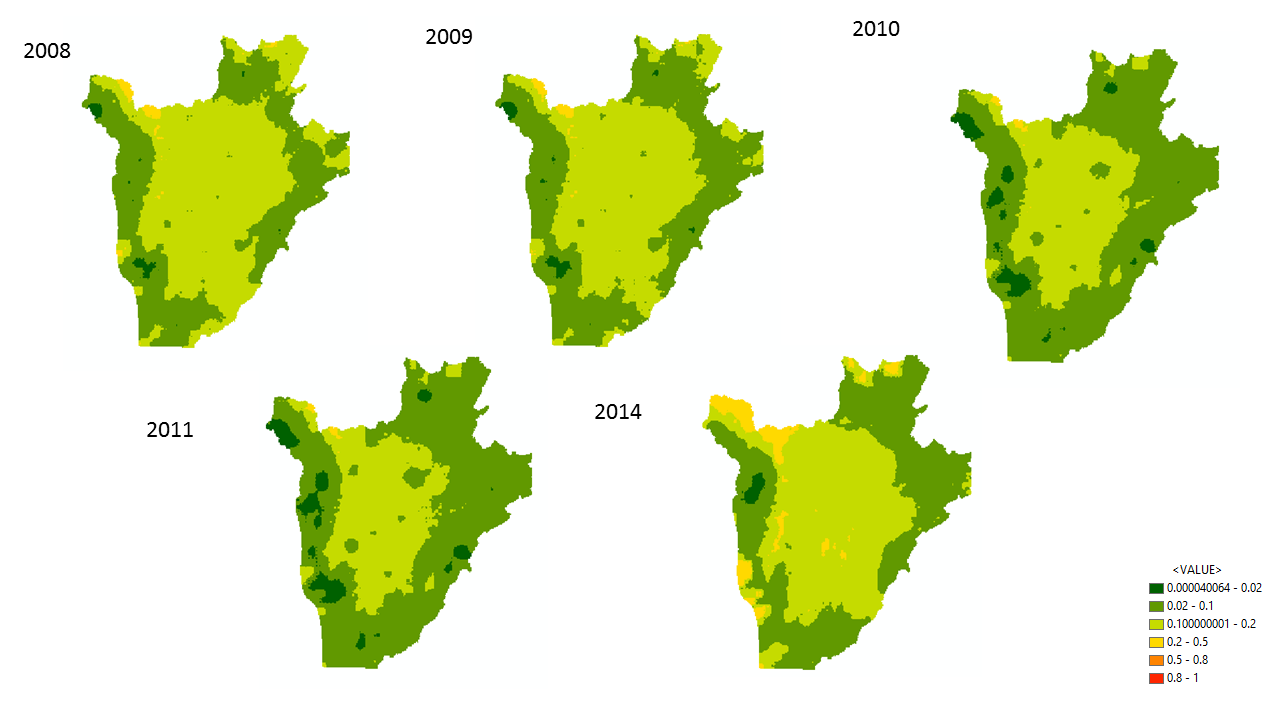


Figure S5: Posterior mean standard deviation of predicted prevalence of infection with *Trichuris Trichiura* in Burundi for 2008 – 2011 and 2014


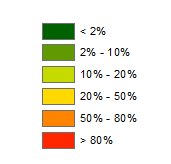

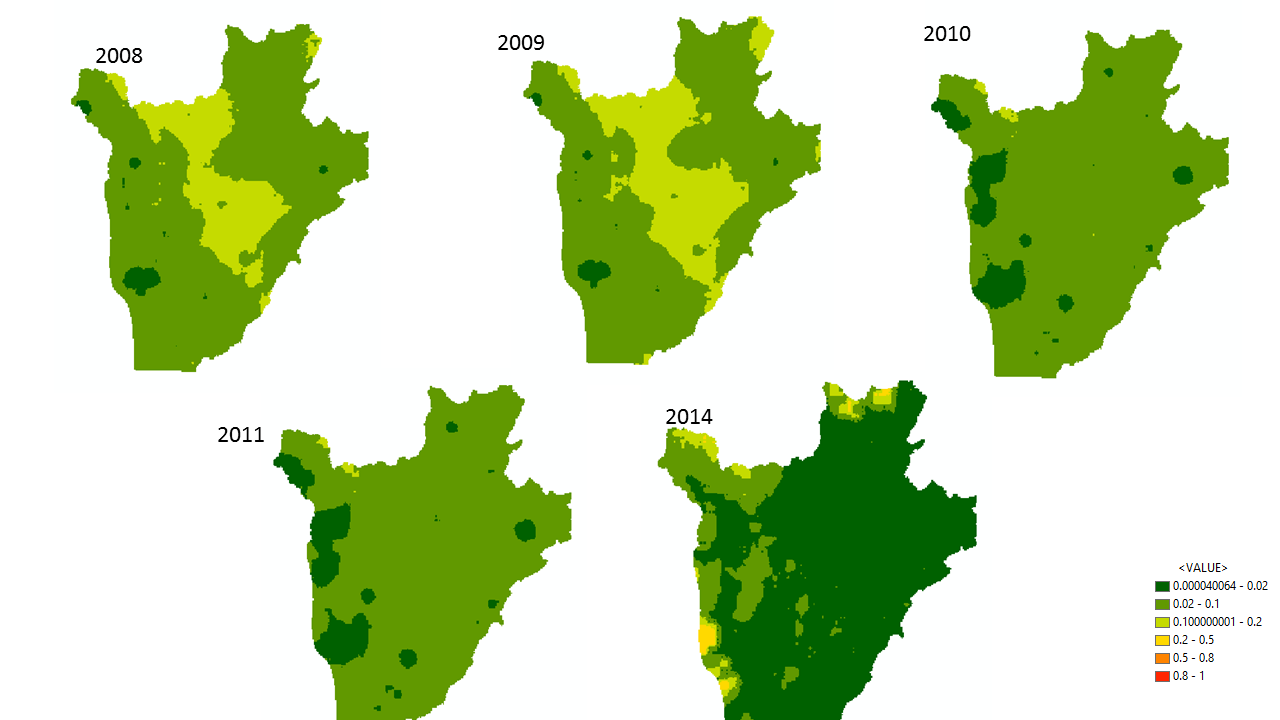


Figure S6: Posterior mean standard deviation of predicted prevalence of infection with *Hookworm* in Burundi for 2008 – 2011 and 2014


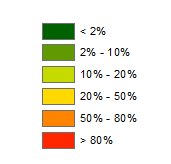

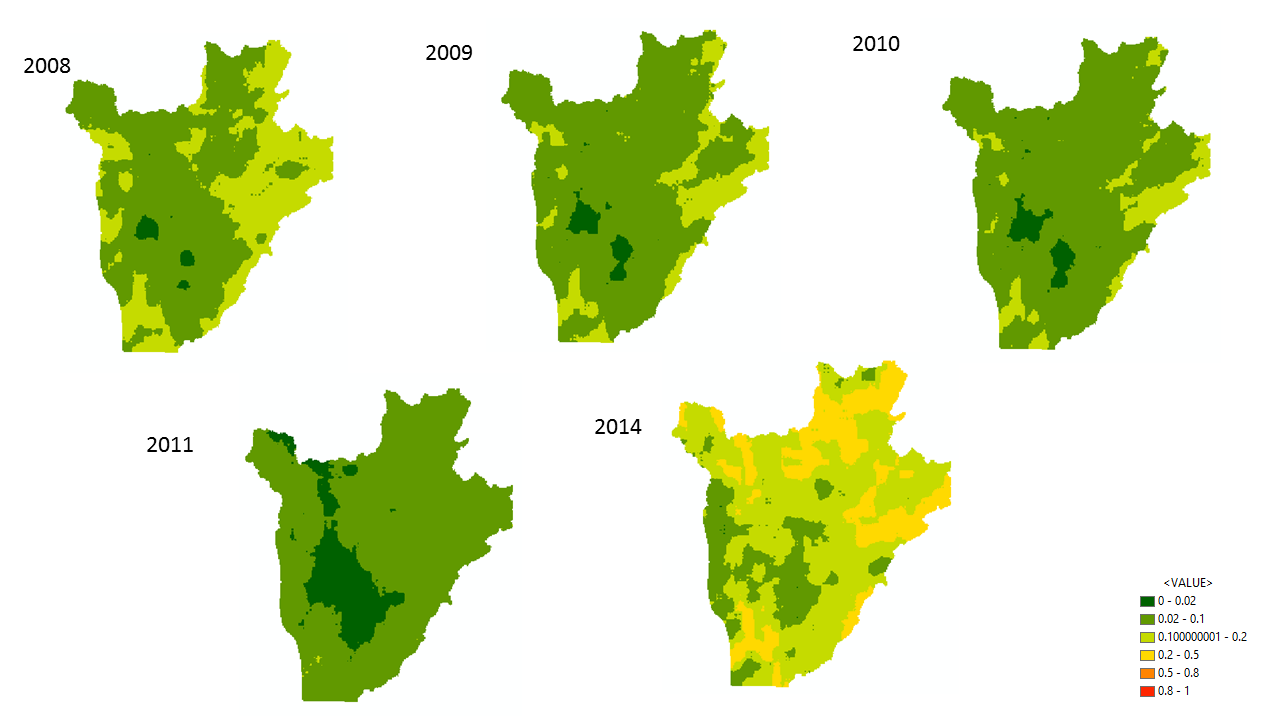


**Table S1: Environmental Data Sumamry Table**

| **Variable** | **Satellite** | **Description** | **Pixel** | **Period** |
| --- | --- | --- | --- | --- |
| Land Surface Temperature | Landsat 5 and Landsat 7 | Obtained from USGS, one image per month over the six years of the study were collected and aggregated into yearly land surface temperature images | 30m x 30m pixels | 2006 – 2014 |
| Elevation | ASTER - GDEM | Advanced Space-borne Thermal Emission and Reflection Radiometer (ASTER) Global Digital Elevation Model (GDEM) | 30m x 30m pixels | 2010 |
| NDVI | Landsat 5 and Landsat 7 | Obtained from USGS, one image per month over the six years of the study were collected and aggregated into normalised difference vegetation index images. | 30m x 30m pixels | 2006 – 2014 |
| Precipitation | WorldClim | WorldClim Precipitation ESRI files | 1km pixels | 2007-2014 |
| Distance to perennial water bodies | Landsat 8 | Collected from a single Landsat 8 image and calculated using nautical distance to sentinel sites | 30m x 30m pixels | 2008 |

**Table S2:** **Correlation Coefficients for NDVI and LST, 2007 - 2011**

|  | NDVI2008 | NDVI2009 | NDVI2010 | NDVI2010 | NDVI2014 |
| --- | --- | --- | --- | --- | --- |
| NDVI2008 | 1 |  |  |  |  |
| NDVI2009 | 0.86 | 1 |  |  |  |
| NDVI2010 | 0.86 | 0.93 | 1 |  |  |
| NDVI2011 | 0.81 | 0.93 | 0.9 | 1 |  |
| NDVI2014 | 0.81 | 0.91 | 0.9 | 0.91 | 1 |
|  | LST2008 | LST2009 | LST2010 | LST2011 | LST2014 |
| LST2008 | 1 |  |  |  |  |
| LST2009 | 0.98 | 1 |  |  |  |
| LST2010 | 0.96 | 0.99 | 1 |  |  |
| LST2011 | 0.94 | 0.98 | 0.98 | 1 |  |
| LST2014 | 0.95 | 0.98 | 0.98 | 0.98 | 1 |

**Table S3: Model Validation results, Mean Error, Mean Absolute Error and Pearson Correlation Coefficient for all parasites, 2008 -2011 and 2014.**

| Year | Mean Error | Mean Absolute Error | MAE/Mean(Obs) | Pearson's Correlation Coefficient |
| --- | --- | --- | --- | --- |
| *A. lumbricoides* | | | | |
| 2008 | 0.11 | 0.06 | 36.15% | 0.84 |
| 2009 | 0.00 | 0.05 | 39.59% | 0.84 |
| 2010 | 0.06 | 0.03 | 22.34% | 0.98 |
| 2011 | -0.04 | 0.03 | 26.11% | 0.91 |
| 2014 | -0.05 | 0.04 | 31.65% | 0.94 |
| *T. trichiura* | | | | |
| 2008 | 0.08 | 0.04 | 53.15% | 0.94 |
| 2009 | 0.02 | 0.03 | 38.27% | 0.93 |
| 2010 | -0.14 | 0.02 | 94.83% | 0.47 |
| 2011 | 0.00 | 0.01 | 41.43% | 0.94 |
| 2014 | 0.00 | 0.03 | 118.44% | 0.16 |
| Hookworm | | | | |
| 2008 | 0.01 | 0.04 | 30.89% | 0.78 |
| 2009 | 0.09 | 0.04 | 38.84% | 0.74 |
| 2010 | -0.35 | 0.03 | 46.47% | 0.83 |
| 2011 | -0.02 | 0.03 | 55.75% | 0.76 |
| 2014 | -0.23 | 0.04 | 58.43% | 0.84 |

**Table S4: Average size of clusters and propensity of clustering per parasite per year.**

| Year | *Ascaris lumbricoides* | *Trichuris trichiura* | Hookworm |
| --- | --- | --- | --- |
| 2007 | None | None | None |
| Propensity for clustering (%) | NA | NA | NA |
| Average size of clusters (km) | NA | NA | NA |
| 2008 | Trended | Clustered |  |
| Propensity for clustering (%) | NA | 100 | 75 |
| Average size of clusters (km) | Spatial trend | 52 | 22 |
| 2009 | None | Clustered | Trended |
| Propensity for clustering (%) | NA | 100 | NA |
| Average size of clusters (km) | NA | 61 | Spatial trend |
| 2010 | Clustered | None | None |
| Propensity for clustering (%) | 80 | NA | NA |
| Average size of clusters (km) | 68 | NA | NA |
| 2011 | Clustered (101km) | Trended | Trended |
| Propensity for clustering (%) | 93 | NA | NA |
| Average size of clusters (km) | 77 | Spatial trend | Spatial trend |
| 2014 | Trended | None | Trended |
| Propensity for clustering (%) | NA | NA | NA |
| Average size of clusters (km) | Spatial trend | NA | Spatial trend |

**Table S5: Model Effect Sizes for all parasites, 2008 -2011 and 2014.**

| Variable | *A. lumbricoides* Mean (95% Cr I) | *T. trichiuris* Mean (95% Cr I) | *Hookworm* Mean (95% Cr I) |
| --- | --- | --- | --- |
|  |  |  |  |
| 2008 - 2011 | | | |
| Location | -2.26 (-2.779, -1.715) | -1.7 (-4.00, 0.65) | -0.78 (-1.88, 1.47) |
| Time 2 - 2008 | 0.21 (0.08, 0.33) | -0.028 (-0.25, 0.20) | -0.85 (-0.98, -0.72) |
| Time 3 - 2009 | 0.07 (-0.05, 0.20) | 0.11 (-0.11, 0.33) | -1.15 (-1.28, -1.02) |
| Time 4 - 2010 | -0.41 (-0.55, -0.27) | -0.73 (-1.01, -0.44) | -1.26 (-1.40, -1.12) |
| Time 5 - 2011 | -0.50 (-0.63, -0.37) | -0.82 (-1.06, -0.58) | -1.91 (-2.05, -1.77) |
| Age 2 (5 -14yrs) | -0.14 (-0.21, -0.07) | -0.038 (-0.14, 0.07) | 0.07 (-0.01, 0.15) |
| Age 3 (>14yrs) | -0.35 (-0.45, -0.24) | -0.26 (-0.42, -0.10) | 0.05 (-0.07, 0.16) |
| Sex | 0.09 (0.03, 0.15) | 0.03 (-0.06, 0.13) | 0.09 (0.02, 0.17) |
| LST | -0.97 (-1.32, -0.56) | -0.35 (-0.88, 0.25) | 0.60 (0.16, 0.94) |
| NDVI | -0.07 (-0.40, 0.31) | 0.09 (-0.32, 0.42) | 0.12 (-0.16, 0.42) |
| ϕ | 7.01 | 3.18 | 7.56 |
| σ | 1.16 | 5.48 | 2.11 |
| τ | 1.08 | 0.5 | 1.18 |
| 2014 | | | |
| Location | -2.94 (-4.19, -1.96) | -3.67 (-4.30, -3.15) | -3.67 (-5.10, -1.47) |
| Sex | 0.17 (-0.21, 0.53) | -0.26 (-0.91, 0.40) | 0.36 (-0.12, 0.86) |
| LST | -0.80 (-1.54, -0.07) | 0.15 (-0.25, 0.58) | -0.16 (-1.33, 0.79) |
| NDVI | 0.30 (-0.61, 1.32) | 0.24 (-0.24, 0.73) | 0.11 (-0.98, 1.28) |
| ϕ | 7.05 | 10.06 | 9.75 |
| σ | 2.23 | 0.19 | 4.2 |
| τ | 0.74 | 178.2 | 0.35 |

**Table S6: Total number of infected children per year per parasite 2008 – 2001 and 2014.**

| District | *Ascaris lumbricoides* | | | | | *Trichuris trichiura* | | | | | *Hookworms* | | | | |
| --- | --- | --- | --- | --- | --- | --- | --- | --- | --- | --- | --- | --- | --- | --- | --- |
|  | 2008 | 2009 | 2010 | 2011 | 2014 | 2008 | 2009 | 2010 | 2011 | 2014 | 2008 | 2009 | 2010 | 2011 | 2014 |
| Bubanza | 43,922 | 41,521 | 30,304 | 29,395 | 38,819 | 18,139 | 21,069 | 10,502 | 9,958 | 9,561 | 44,369 | 36,665 | 34,877 | 20,872 | 59,987 |
| Buhiga | 83,810 | 79,084 | 57,036 | 55,156 | 59,539 | 33,425 | 38,734 | 19,579 | 18,545 | 7,589 | 51,991 | 42,229 | 39,962 | 23,200 | 56,383 |
| Bururi | 36,657 | 34,647 | 25,157 | 24,366 | 35,862 | 9,877 | 11,519 | 5,632 | 5,324 | 4,060 | 16,223 | 13,112 | 12,391 | 7,156 | 20,261 |
| Busoni | 22,275 | 20,860 | 14,783 | 14,273 | 18,730 | 13,626 | 15,815 | 7,931 | 7,511 | 16,292 | 24,034 | 19,509 | 18,462 | 10,732 | 30,624 |
| Butezi | 43,253 | 40,713 | 29,142 | 28,144 | 47,805 | 29,334 | 33,689 | 17,863 | 17,000 | 5,454 | 37,901 | 31,146 | 29,591 | 17,523 | 31,491 |
| Buye | 56,557 | 53,291 | 38,268 | 36,983 | 36,236 | 42,710 | 48,839 | 26,440 | 25,230 | 6,369 | 35,680 | 28,967 | 27,409 | 15,920 | 60,238 |
| Cankuzo | 17,093 | 15,856 | 10,830 | 10,383 | 9,591 | 8,205 | 9,567 | 4,687 | 4,429 | 3,547 | 30,803 | 25,450 | 24,217 | 14,476 | 28,355 |
| Cibitoke | 16,669 | 15,319 | 10,164 | 9,712 | 28,435 | 12,408 | 14,536 | 6,919 | 6,535 | 15,526 | 53,541 | 43,958 | 41,732 | 24,688 | 38,085 |
| Fota | 84,102 | 80,969 | 62,536 | 61,166 | 64,095 | 23,296 | 26,903 | 13,831 | 13,121 | 6,942 | 12,435 | 9,836 | 9,237 | 5,150 | 15,332 |
| Gahombo | 64,888 | 61,629 | 45,421 | 44,080 | 47,182 | 38,119 | 43,620 | 23,535 | 22,434 | 6,245 | 31,395 | 25,519 | 24,154 | 14,059 | 45,937 |
| Gashoho | 27,873 | 25,958 | 17,944 | 17,238 | 17,926 | 17,431 | 20,223 | 10,149 | 9,612 | 4,701 | 31,406 | 25,655 | 24,326 | 14,255 | 51,845 |
| Gihofi | 30,765 | 28,745 | 20,085 | 19,328 | 27,168 | 17,532 | 20,354 | 10,187 | 9,645 | 5,187 | 35,048 | 28,777 | 27,326 | 16,173 | 32,688 |
| Gitega | 119,619 | 114,694 | 87,202 | 85,044 | 112,288 | 60,269 | 68,903 | 37,275 | 35,529 | 8,882 | 47,982 | 38,934 | 36,844 | 21,331 | 22,484 |
| Giteranyi | 38,172 | 35,572 | 24,676 | 23,720 | 18,180 | 25,823 | 29,878 | 15,242 | 14,470 | 6,107 | 51,400 | 42,254 | 40,155 | 23,833 | 65,547 |
| Isale | 53,531 | 50,184 | 35,585 | 34,342 | 34,985 | 24,826 | 28,975 | 14,110 | 13,335 | 14,894 | 59,969 | 48,911 | 46,349 | 27,143 | 34,573 |
| Kabezi | 41,345 | 39,009 | 28,200 | 27,293 | 32,431 | 15,563 | 18,132 | 8,897 | 8,412 | 7,110 | 29,319 | 23,935 | 22,689 | 13,309 | 18,718 |
| Kayanza | 114,723 | 109,669 | 82,837 | 80,759 | 122,551 | 52,932 | 60,609 | 32,617 | 31,103 | 24,275 | 22,139 | 17,499 | 16,423 | 9,147 | 45,210 |
| Kibumbu | 91,983 | 88,605 | 68,433 | 66,915 | 64,786 | 28,868 | 33,292 | 17,194 | 16,317 | 6,162 | 16,182 | 12,850 | 12,080 | 6,771 | 14,472 |
| Kibuye | 111,749 | 106,755 | 80,261 | 78,144 | 128,903 | 61,315 | 70,025 | 38,165 | 36,430 | 9,484 | 31,852 | 25,520 | 24,053 | 13,664 | 31,906 |
| Kiganda | 70,536 | 67,541 | 51,252 | 49,989 | 52,552 | 24,972 | 28,784 | 14,962 | 14,211 | 5,776 | 21,481 | 17,237 | 16,254 | 9,259 | 21,944 |
| Kinyinya | 17,041 | 15,743 | 10,610 | 10,151 | 22,247 | 17,963 | 20,850 | 10,461 | 9,904 | 6,605 | 56,467 | 47,280 | 45,186 | 27,643 | 37,529 |
| Kiremba | 60,049 | 56,231 | 39,606 | 38,163 | 41,122 | 39,063 | 45,089 | 23,252 | 22,088 | 8,458 | 54,442 | 44,309 | 41,967 | 24,463 | 87,511 |
| Kirundo | 18,964 | 17,685 | 12,373 | 11,923 | 22,605 | 13,620 | 15,874 | 7,784 | 7,355 | 22,710 | 26,452 | 21,345 | 20,161 | 11,586 | 40,440 |
| LAKE | 14,567 | 13,400 | 8,946 | 8,553 | 18,432 | 10,888 | 12,755 | 6,092 | 5,746 | 18,430 | 42,567 | 34,950 | 33,191 | 19,681 | 16,620 |
| Mabayi | 45,640 | 43,091 | 31,372 | 30,442 | 109,086 | 20,785 | 24,012 | 12,359 | 11,757 | 35,302 | 25,246 | 20,201 | 19,027 | 10,812 | 45,966 |
| Makamba | 46,373 | 43,287 | 30,170 | 29,022 | 29,925 | 23,302 | 27,080 | 13,491 | 12,769 | 6,425 | 46,261 | 37,639 | 35,643 | 20,768 | 34,484 |
| Matana | 107,618 | 102,887 | 77,471 | 75,458 | 92,387 | 20,854 | 24,291 | 11,920 | 11,276 | 8,997 | 12,125 | 9,519 | 8,914 | 4,918 | 17,640 |
| Mpanda | 23,675 | 22,120 | 15,528 | 14,967 | 13,871 | 10,005 | 11,708 | 5,624 | 5,311 | 7,873 | 38,032 | 31,097 | 29,475 | 17,305 | 25,671 |
| Mukenke | 18,096 | 16,792 | 11,493 | 11,024 | 9,816 | 14,314 | 16,597 | 8,363 | 7,928 | 3,943 | 28,854 | 23,651 | 22,452 | 13,260 | 42,821 |
| Muramvya | 63,376 | 60,672 | 46,011 | 44,875 | 51,276 | 18,106 | 20,945 | 10,678 | 10,124 | 7,113 | 12,054 | 9,542 | 8,960 | 5,000 | 22,147 |
| Murore | 17,879 | 16,644 | 11,505 | 11,051 | 10,659 | 10,589 | 12,282 | 6,189 | 5,861 | 3,411 | 32,723 | 27,227 | 25,968 | 15,724 | 30,293 |
| Musema | 106,111 | 101,164 | 75,524 | 73,450 | 81,952 | 40,827 | 47,111 | 24,346 | 23,106 | 10,212 | 36,602 | 29,446 | 27,782 | 15,889 | 47,036 |
| Mutaho | 85,842 | 81,802 | 61,005 | 59,321 | 75,644 | 35,494 | 40,903 | 21,296 | 20,225 | 6,530 | 43,559 | 35,502 | 33,634 | 19,633 | 32,110 |
| Muyinga | 48,273 | 44,957 | 31,087 | 29,861 | 24,854 | 26,532 | 30,849 | 15,323 | 14,492 | 7,428 | 66,628 | 55,004 | 52,325 | 31,200 | 65,793 |
| Ngozi | 100,485 | 95,238 | 69,686 | 67,543 | 67,407 | 65,669 | 74,990 | 40,872 | 39,010 | 9,354 | 48,250 | 39,132 | 37,014 | 21,464 | 74,470 |
| Nyabikere | 85,289 | 80,745 | 58,835 | 56,977 | 75,162 | 32,647 | 37,792 | 19,236 | 18,221 | 7,384 | 49,707 | 40,554 | 38,434 | 22,489 | 34,640 |
| Nyanza-Lac | 25,997 | 24,091 | 16,403 | 15,720 | 16,263 | 18,869 | 21,995 | 10,776 | 10,182 | 4,021 | 49,925 | 41,323 | 39,338 | 23,596 | 53,462 |
| Rumonge | 40,853 | 38,345 | 27,235 | 26,289 | 42,114 | 11,604 | 13,616 | 6,447 | 6,082 | 7,707 | 53,015 | 43,560 | 41,365 | 24,533 | 53,342 |
| Rutana | 53,768 | 50,650 | 36,324 | 35,099 | 66,788 | 35,108 | 40,364 | 21,191 | 20,148 | 7,019 | 23,300 | 18,666 | 17,594 | 9,996 | 27,546 |
| Ruyigi | 22,551 | 21,104 | 14,825 | 14,275 | 21,062 | 14,857 | 17,135 | 8,895 | 8,448 | 3,203 | 28,954 | 23,987 | 22,845 | 13,722 | 31,113 |
| Rwibaga | 60,120 | 57,513 | 43,559 | 42,480 | 58,575 | 13,658 | 15,851 | 7,942 | 7,523 | 9,480 | 7,566 | 5,954 | 5,583 | 3,091 | 12,459 |
| Ryansoro | 79,507 | 76,647 | 59,469 | 58,211 | 73,578 | 28,593 | 32,822 | 17,389 | 16,551 | 6,104 | 12,639 | 10,022 | 9,419 | 5,270 | 10,887 |
| Vumbi | 18,408 | 16,983 | 11,407 | 10,912 | 15,624 | 18,119 | 21,067 | 10,444 | 9,879 | 6,895 | 34,022 | 27,599 | 26,109 | 15,129 | 64,438 |
| ZONE-Centre | 3,385 | 3,098 | 2,031 | 1,935 | 1,748 | 2,672 | 3,138 | 1,480 | 1,393 | 2,692 | 9,510 | 7,720 | 7,305 | 4,235 | 1,830 |
| ZONE-Nord | 10,681 | 9,773 | 6,395 | 6,091 | 4,487 | 9,763 | 11,458 | 5,419 | 5,105 | 7,213 | 29,887 | 24,290 | 22,988 | 13,376 | 9,648 |
| ZONE-Sud | 5,105 | 4,671 | 3,059 | 2,913 | 2,674 | 3,576 | 4,208 | 1,970 | 1,853 | 4,199 | 17,293 | 14,161 | 13,439 | 7,901 | 2,546 |
| Total | 2,349,174 | 2,225,952 | 1,632,045 | 1,583,134 | 2,047,423 | 1,116,149 | 1,288,251 | 664,961 | 631,491 | 402,870 | 1,571,231 | 1,281,640 | 1,214,649 | 711,346 | 1,648,523 |
